# Repurposing loratadine to induce ferroptosis and overcome multidrug resistance: preclinical evidence in KB-V-1 cells

**DOI:** 10.3389/fonc.2026.1829405

**Published:** 2026-06-17

**Authors:** Nicholas Cook, Xin Li, Nathan J. Bowen, Alira Danaher, MaKayla Thomas, Saani Alhassan, Briah Arms, Daqing Wu

**Affiliations:** 1 Center for Cancer Research and Therapeutic Development and Department of Biological Sciences, Clark Atlanta University, Atlanta, GA, United States; 2 MetCure Therapeutics LLC, Atlanta, GA, United States

**Keywords:** drug repurposing, ER stress, ferroptosis, loratadine, multidrug resistance, SLC7A11

## Abstract

**Background:**

MDR1/P-glycoprotein-mediated multidrug resistance (MDR) limits chemotherapy efficacy across many cancers. Ferroptosis, an iron-dependent regulated cell death driven by lipid peroxidation, offers a potential strategy to bypass MDR. We investigated whether loratadine, an FDA-approved antihistamine, induces ferroptosis and overcomes chemoresistance in MDR1-overexpressing KB-V-1 cells.

**Methods:**

Cytotoxicity was evaluated using the CCK-8 assay in multidrug-resistant KB-V-1 cells and chemosensitive KB-3–1 cells. Ferroptosis was verified using specific inhibitors, including ferrostatin-1, liproxstatin-1, and deferoxamine, and compared with the pan-caspase inhibitor Z-VAD-FMK to exclude apoptosis. Hallmarks of ferroptosis (cystine uptake, intracellular glutathione levels, labile iron pool, and lipid peroxidation) were quantitatively assessed. Transcriptomic alterations were analyzed by RNA sequencing (RNA-seq) and further validated by quantitative real-time PCR (qRT-PCR) and Western blot analyses. *In vivo* antitumor efficacy was examined in a KB-V-1 xenograft model following treatment with loratadine (20 mg/kg, intraperitoneal), paclitaxel, or their combination.

**Results:**

Loratadine selectively inhibited KB-V-1 viability (IC_50_ = 2.0 µM) while sparing KB-3–1 cells (IC_50_ =48.45 µM). Cell death was effectively rescued by ferroptosis inhibitors, confirming ferroptosis as the primary mechanism. Loratadine suppressed cystine uptake, depleted glutathione, elevated labile iron, and promoted lipid peroxidation comparably to erastin. RNA-seq identified 1,861 differentially expressed genes enriched in the PERK-eIF2α-ATF4 stress axis; upregulation of ATF4, CHAC1, DDIT4, and DDIT3 was confirmed by qRT-PCR and/or Western blot analyses. Loratadine also reduced MDR1/P-glycoprotein expression. *In vivo*, loratadine suppressed tumor growth and enhanced paclitaxel efficacy without systemic toxicity.

**Conclusion:**

Loratadine induces ferroptosis in MDR1-overexpressing cells via cystine uptake inhibition, glutathione depletion, and PERK-eIF2α-ATF4 activation, while concurrently downregulating MDR1/P-glycoprotein. These findings support loratadine as a clinically actionable repurposing candidate for ferroptosis-based, resistance-directed cancer therapy.

## Introduction

Chemotherapy remains a cornerstone of cancer treatment; however, its long-term efficacy is frequently limited by the emergence of multidrug resistance, resulting in treatment failure and disease recurrence. Overexpression of the ATP-binding cassette transporter ABCB1 (MDR1/P-glycoprotein) is a well-established mechanism of resistance that reduces intracellular accumulation of structurally diverse chemotherapeutic agents, including taxanes and vinca alkaloids. Despite decades of investigation, clinically effective strategies to overcome multidrug resistance remain limited, underscoring the need for alternative therapeutic approaches that exploit vulnerabilities unique to resistant cancer cells (1–3).

Ferroptosis, an iron-dependent form of regulated cell death driven by lipid peroxidation, has emerged as a promising strategy to circumvent therapeutic resistance (4). Central to ferroptosis regulation is the cystine/glutamate antiporter SLC7A11 (xCT), which imports cystine to sustain glutathione (GSH) synthesis. GSH serves as an essential cofactor for glutathione peroxidase 4 (GPX4), the primary enzyme responsible for detoxifying lipid hydroperoxides. Inhibition of SLC7A11 depletes intracellular GSH, impairs GPX4 activity, thereby promoting lethal lipid peroxide accumulation (5–7). Notably, drug-resistant cancer cells frequently exhibit altered redox homeostasis and elevated oxidative stress tolerance, creating a potential vulnerability to ferroptosis induction (8). Thus, therapeutically disrupting antioxidant defenses represents a compelling strategy for targeting chemoresistant tumors.

Beyond the canonical SLC7A11-GSH-GPX4 axis, ferroptosis is tightly integrated with endoplasmic reticulum (ER) stress signaling. Inhibition of cystine uptake activates the protein kinase RNA-like endoplasmic reticulum kinase (PERK)-eukaryotic translation initiation factor 2α (eIF2α)-activating transcription factor 4 (ATF4) branch of the unfolded protein response, inducing stress-responsive genes such as glutathione-specific γ-glutamylcyclotransferase 1 (CHAC1), DNA damage-inducible transcript 4 (DDIT4), C/EBP homologous protein (CHOP/DDIT3), tribbles homolog 3 (TRIB3), and asparagine synthetase (ASNS), which collectively amplify redox imbalance and ferroptotic signaling (9, 10). This convergence of ER stress and ferroptosis suggests that activation of the integrated stress response may lower the threshold for ferroptotic cell death in therapy-resistant malignancies.

Drug repurposing offers a cost- and time-efficient strategy to accelerate the development of new anticancer therapies. Among repurposing candidates, antihistamines have attracted attention for potential antitumor activity. Loratadine (commonly known as Claritin), a second-generation histamine H1 receptor antagonist widely prescribed for allergic disorders, has demonstrated antiproliferative effects across multiple cancer models (9, 11–15). Reported mechanisms include induction of DNA damage, modulation of signal transducer and activator of transcription 3 (STAT3) signaling, and activation of apoptosis or pyroptosis (11, 16–18). Notably, a systematic screen of 21 histamine receptor antagonists revealed that only loratadine and azelastine were capable of highly sensitizing MDR1/P-glycoprotein-overexpressing KBV20C cells to antimitotic agents such as vincristine and eribulin, and this sensitizing activity was found to be independent of histamine receptor inhibitory function per se (19). However, most studies have been conducted in drug-sensitive settings, and the impact of loratadine on multidrug resistant tumors remains poorly defined. Moreover, the molecular basis underlying its anticancer activity has not been fully elucidated.

We recently identified loratadine as a potent inhibitor of chemoresistant prostate cancer cell growth through suppression of SLC7A11 and induction of ferroptosis (manuscript under revision) (20). These findings raised the possibility that loratadine may exploit ferroptotic vulnerabilities inherent to resistant cancer cells. To determine whether this mechanism extends beyond prostate cancer and applies to classical MDR1-overexpressing models, we investigated the effects of loratadine in KB-V-1 cells, a multidrug resistant derivative of the KB-3–1 human carcinoma line selected with vinblastine. KB-V-1 cells overexpress MDR1/P-glycoprotein and exhibit broad cross-resistance to multiple cytotoxic agents, including paclitaxel (21–24), making them a well-characterized model for evaluating strategies to overcome P-glycoprotein-mediated resistance.

In this study, we demonstrate that loratadine selectively induces ferroptosis in MDR1-overexpressing KB-V-1 cells and suppresses MDR1 expression. *In vivo*, loratadine significantly suppressed tumor growth and enhanced paclitaxel efficacy in a KB-V-1 xenograft model without overt toxicity. Together, these findings identify loratadine as a repurposed agent that targets both redox homeostasis and drug efflux pathways, providing a translational framework for overcoming multidrug resistance through ferroptosis activation.

## Materials and methods

### Cell lines and chemicals

Chemosensitive KB-3–1 cells and multidrug resistant KB-V-1 cells were kindly provided by Dr. Georgia Z. Chen (Emory University, Atlanta, GA) (21–24). KB-3–1 cells were maintained in DMEM medium (Fisher Scientific, Hampton, NH) supplemented with 10% fetal bovine serum (VWR, Radnor, PA) and 1% penicillin/streptomycin (Thermo Fisher Scientific Inc., Waltham, MA). KB-V-1 cells were maintained in DMEM supplemented with 10% fetal bovine serum, 1% penicillin/streptomycin, and 0.5 μg/ml vinblastine (Thermo Fisher). Dimethyl sulfoxide (DMSO) and deferoxamine (DFO) were purchased from Thermo Fisher. Erastin and liproxstatin-1 (Lip-1) were purchased from Selleck Chemicals LLC (Houston, TX). Loratadine, paclitaxel, ferrostatin-1 (Fer-1), and polyethylene glycol 400 (PEG 400) were purchased from Sigma-Aldrich (St. Louis, MO). Z-VAD-FMK was purchased from Santa Cruz Biotechnology Inc (Dallas, TX).

### *In vitro* cytotoxicity assay

Cell viability was assessed using the Cell Counting Kit-8 (CCK-8; Dojindo Molecular Technologies, Rockville, MD) according to the manufacturer’s instructions. KB-V-1 cells were treated with increasing concentrations of loratadine or erastin (0-100 µM), and absorbance was measured to determine cell viability. Half-maximal inhibitory concentrations (IC_50_) were calculated using GraphPad Prism software (Dotmatics, San Diego, CA).

### Cystine uptake analysis

Cystine uptake was measured using the Cystine Uptake Assay Kit (Dojindo) following the manufacturer’s protocol. KB-V-1 cells were incubated with 10 µM loratadine, 10 µM erastin, or vehicle control in serum- and cystine-free DMEM for 1 h at 37 °C in a 5% CO_2_ incubator. After removing the medium, cells were exposed to prewarmed CA uptake solution (DMEM containing 10 µM loratadine, 10 µM erastin, or vehicle) for an additional 1.5 h under the same conditions. Cells were then washed three times with cold PBS, followed by the addition of methanol and working solution with gentle mixing. Plates were incubated for 30 min, and fluorescence was measured using a BioTek Synergy H1 multimode reader (Agilent Technologies, Inc. Santa Clara, CA) with Ex/Em settings of 490/535 nm. Values were background-subtracted and normalized to the vehicle control group.

### Intracellular glutathione assay

Intracellular GSH levels were measured using the GSH-Glo™ Glutathione Assay (V6912, Promega, Madison, WI) according to the manufacturer’s instructions. KB-V-1 cells were treated with loratadine (5 µM), erastin (5 µM), or vehicle control for 24 h at 37°C in a 5% CO_2_ incubator. After treatment, the medium was removed, and cells were incubated with 1× GSH-Glo Reagent at room temperature for 30 min, followed by addition of Luciferin Detection Reagent and incubation for 15 min. Luminescence was measured using a BioTek Synergy H1 multimode reader. GSH levels were quantified using a GSH standard curve.

### Intracellular iron assay

Intracellular iron levels were assessed using FerroOrange (Dojindo, F374). A 1 mM FerroOrange stock solution was prepared in DMSO and diluted with serum-free culture medium to a final concentration of 1 µM. KB-V-1 cells were seeded in black 96-well plates and treated with loratadine (5 µM), erastin (5 µM), or vehicle control for 24 h. Following treatment, the medium was removed, and cells were washed three times with HBSS. Cells were then incubated with 1 µM FerroOrange working solution for 30 min at 37 °C in a 5% CO_2_ incubator. Fluorescence was measured using a BioTek Synergy H1 multimode reader (Ex/Em = 543/580 nm). Intracellular iron levels were quantified according to the manufacturer’s instructions and normalized to total protein content.

### Lipid peroxidation

KB-V-1 cells were treated with loratadine (5 µM), erastin (5 µM), or vehicle control for 24 h at 37 °C in a 5% CO_2_ incubator. After treatment, cells were washed twice with HBSS and incubated with BODIPY™ 581/591 C11 (BDP 581/591 C11; Dojindo, L267) working solution for 30 min at 37 °C. Cells were then washed twice with HBSS and incubated in HBSS containing stimulation agents for an additional 2 h at 37 °C. Following incubation, cells were washed twice with HBSS, and fluorescence was measured using a BioTek Synergy H1 multimode reader (green: Ex/Em = 500/540 nm; red: Ex/Em = 550/610 nm). Lipid peroxidation was quantified as the ratio of red to green fluorescence and normalized to the vehicle control group.

### Real-time quantitative PCR

Total RNA was isolated using the Quick-RNA MiniPrep Plus kit (R1057, Zymo Research, Irvine, CA). First-strand cDNA was synthesized from 1 µg of total RNA using oligo(dT) primers (E6560, New England BioLabs, Ipswich, MA) following the manufacturer’s instructions. qRT-PCR was performed using PowerUp SYBR Green Master Mix (4367659, Applied Biosystems, Waltham, MA) on a Bio-Rad CFX Opus 96 Real-Time PCR System (Bio-Rad Laboratories, Hercules, CA). Gene expression levels were normalized to glyceraldehyde-3-phosphate dehydrogenase (GAPDH), and relative expression was calculated using Bio-Rad analysis software. Primers used in this study are listed in **Supplementary Table 1**.

### RNA sequencing analysis

RNA samples were collected from KB-V-1 cells treated with vehicle control (DMSO) or loratadine (2.0 μM) for 24 h, with three biological replicates per condition (n = 6 total samples). Total RNA was submitted to Omega Bioservices (Norcross, GA), where poly(A)+ RNA enrichment, stranded library preparation (NEBNext Poly(A) mRNA Magnetic Isolation Module, E7490L; and NEBNext Ultra II Directional RNA Library Prep Kit for Illumina, E7760L), and sequencing were performed. Libraries were sequenced on an Illumina NovaSeq X Plus platform using paired-end 150-bp reads (2 x 150 bp; reverse-stranded RNA-seq).

Downstream biological interpretation was performed using Ingenuity Pathway Analysis (IPA; Qiagen) and Gene Set Enrichment Analysis (GSEA; UC San Diego/Broad Institute) (25–27). Functional enrichment analysis was also performed using WebGestalt and WebGestaltR (28, 29).

### Western blot analysis

Total cell lysates were prepared using radioimmunoprecipitation assay (RIPA) buffer supplemented with Halt™ phosphatase and protease inhibitor cocktail (Thermo Fisher). Protein concentrations were determined using the Pierce BCA Protein Assay Kit (23225, Thermo Fisher). Samples were mixed with 4× LDS Sample Buffer (GenScript, Piscataway, NJ) and boiled at 95 °C before electrophoresis. The antibodies used are listed in **Supplementary Table 2**. All Western blot analyses were repeated at least three times.

### *In vivo* efficacy studies

All animal procedures were approved by the Morehouse School of Medicine Institutional Animal Care and Use Committee and conducted in accordance with National Institutes of Health guidelines. KB-V-1 cells (1 × 10^6^ per site) were mixed 1:1 (v/v) with Matrigel HC (Corning Inc., Corning, NY) and injected subcutaneously into male athymic nude mice (5-week-old; Envigo RMS, Inc., Indianapolis, IN). When tumors reached a mean volume of approximately ≤ 100 mm³, tumor-bearing mice were randomized into the following treatment groups: vehicle control (10% DMSO, 40% ethanol, and 50% PEG 400; v/v/v), paclitaxel (10 mg/kg, once weekly), loratadine (20 mg/kg, three times weekly), or a combination of paclitaxel and loratadine. Treatments were administered via intraperitoneal injection. Dose selection for loratadine was based on its single-agent efficacy without apparent toxicity in our previous xenograft models (20). The paclitaxel dose was selected based on reported therapeutic doses used in similar xenograft models (30, 31), together with our pilot studies in the KB-V-1 model. Tumor growth was measured three times weekly using digital calipers. Tumor volume was calculated using the formula V = (L × W²)/2, where L is the longest tumor diameter and W is the shorter diameter perpendicular to L. Body weight and animal behavior were also monitored three times weekly. A humane endpoint of 15 days was used when tumors in control animals approached the maximum allowable size (15 mm in any dimension).

### Statistical analysis

All *in vitro* data represent three or more experiments. The unpaired *t*-test was used to determine the significance of differences between any two groups. Two-way analysis of variance (ANOVA) was used to test the overall difference between different treatment groups during the whole study period. GraphPad Prism software (Dotmatics, San Diego, CA) was used to perform the statistical analyses. *p* < 0.05 was considered statistically significant. Combination antitumor effects were quantified using the Bliss independence model (32). Tumor growth inhibition (TGI) for each treatment arm was calculated as: TGI = 1 − (mean tumor volume of treated group/mean tumor volume of vehicle control). The expected additive TGI under the Bliss model was determined by the formula: TGI_Expected = TGI_A + TGI_B − (TGI_A × TGI_B), where TGI_A and TGI_B represent the inhibition values for the individual agents. The Bliss Excess Score was calculated as the difference between the observed and expected TGI; a positive value indicates supra-additivity and a score ≥ 10% indicates synergism. Tumor burden over the treatment period was additionally quantified as a supplementary measure by calculating the area under the tumor growth curve (AUC) using the trapezoidal method. Briefly, AUC was computed as the sum of trapezoids formed between consecutive measurement time points: AUC = Σ [(t_{i+1} − t_i) × (V_i + V_{i+1})/2], where t denotes the day of measurement and V denotes the mean tumor volume (mm³) at each time point. AUC values were calculated for each treatment group and compared using one-way ANOVA with GraphPad Prism software.

## Results

### Multidrug resistant KB-V-1 cells are susceptible to ferroptosis induction

**Figure 1:**
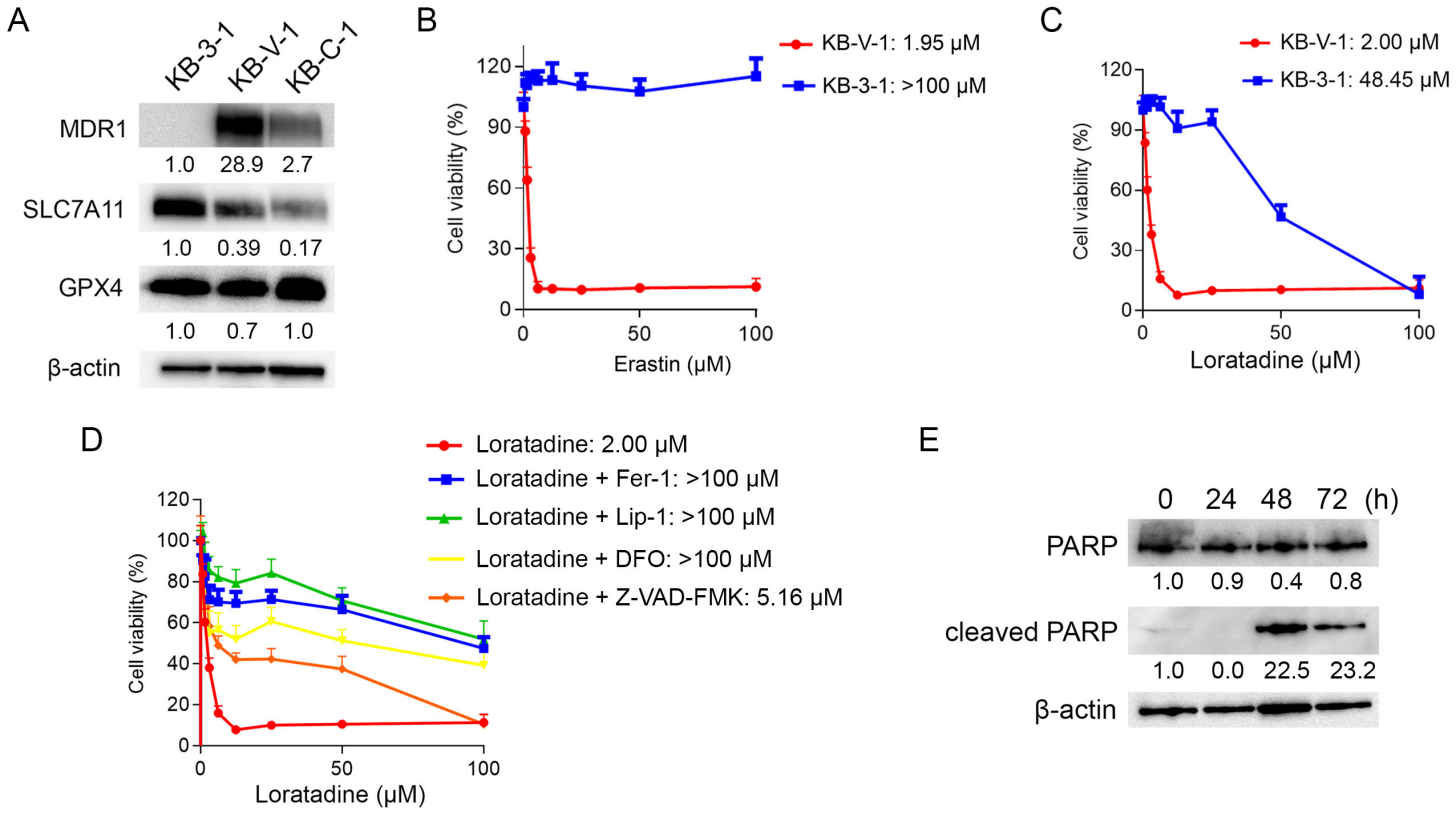
Selective induction of cell death in chemoresistant KB-V-1 cells by loratadine. **(A)** Western blot analysis comparing basal expression levels of MDR1, SLC7A11 and GPX4 in chemosensitive KB-3–1 and chemoresistant KB-V-1 cells. **(B)** *In vitro* cytotoxicity of erastin in KB-V-1 and KB-3–1 cells after 72 h of treatment. **(C)** *In vitro* cytotoxicity of loratadine in KB-V-1 and KB-3–1 cells after 72 h of treatment. **(D)** *In vitro* cytotoxicity of loratadine in KB-V-1 cells in the presence or absence of the ferroptosis inhibitors Fer-1 and Lip-1, the iron chelator DFO, and the caspase inhibitor Z-VAD-FMK following 72 h of treatment. **(E)** Western blot analysis of PARP and cleaved PARP in KB-V-1 cells treated with loratadine (2 µM) for 0–72 h.

To assess whether KB-V-1 cells are prone to ferroptosis, we analyzed the basal expression of key ferroptosis-regulating proteins by Western blot analysis. The results confirmed overexpression of MDR1/P-glycoprotein in KB-V-1 cells relative to no expression in KB-3–1 cells and low expression in KB-C-1 cells. KB-V-1 cells showed reduced SLC7A11 expression compared with KB-3–1 cells, indicating a diminished capacity for cystine uptake and GSH biosynthesis. In contrast, GPX4 was expressed in all KB cells, reflecting a GSH-dependent antioxidant defense (**Figure 1A**). Consistently, treatment with erastin, a well-characterized SLC7A11 inhibitor, resulted in a half-maximal inhibitory concentration (IC_50_) of 1.95 µM in KB-V-1 cells, whereas KB-3–1 cells remained largely resistant (IC_50_ > 100 µM) (**Figure 1B**), indicating that KB-V-1 cells are more susceptible to ferroptosis induction than their chemosensitive counterparts.

### Loratadine induces ferroptosis in KB-V-1 cells

We evaluated the cytotoxic effect of loratadine in both KB-V-1 and KB-3–1 cells. Loratadine exhibited potent inhibitory activity against KB-V-1 cells, with an IC50 of 2.0 µM, whereas the IC50 in KB-3–1 cells as 48.45 µM (**Figure 1C**). To determine whether loratadine-induced cytotoxicity involves ferroptosis, KB-V-1 cells were pretreated with established ferroptosis inhibitors, specifically ferrostatin-1 (Fer-1, a lipid peroxidation scavenger), deferoxamine (DFO, an iron chelator), and liproxstatin-1 (Lip-1, a lipophilic antioxidant that prevents lipid peroxide accumulation), and a caspase inhibitor (Z-VAD-FMK), prior to loratadine exposure. All three ferroptosis inhibitors markedly restored cell viability, whereas the caspase inhibitor Z-VAD-FMK provided only partial protection, shifting the IC_50_ from 2.0 μM to 5.16 μM (**Figure 1D**). To further assess the contribution of apoptosis, we performed Western blot analysis for cleaved poly(ADP-ribose) polymerase (PARP), a hallmark of caspase-dependent apoptosis. Loratadine treatment induced detectable PARP cleavage in KB-V-1 cells (**Figure 1E**). Together, these data indicate that ferroptosis is the predominant mechanism of loratadine-induced cell death, with a modest apoptotic component contributing to the overall cytotoxic response.

**Figure 2:**
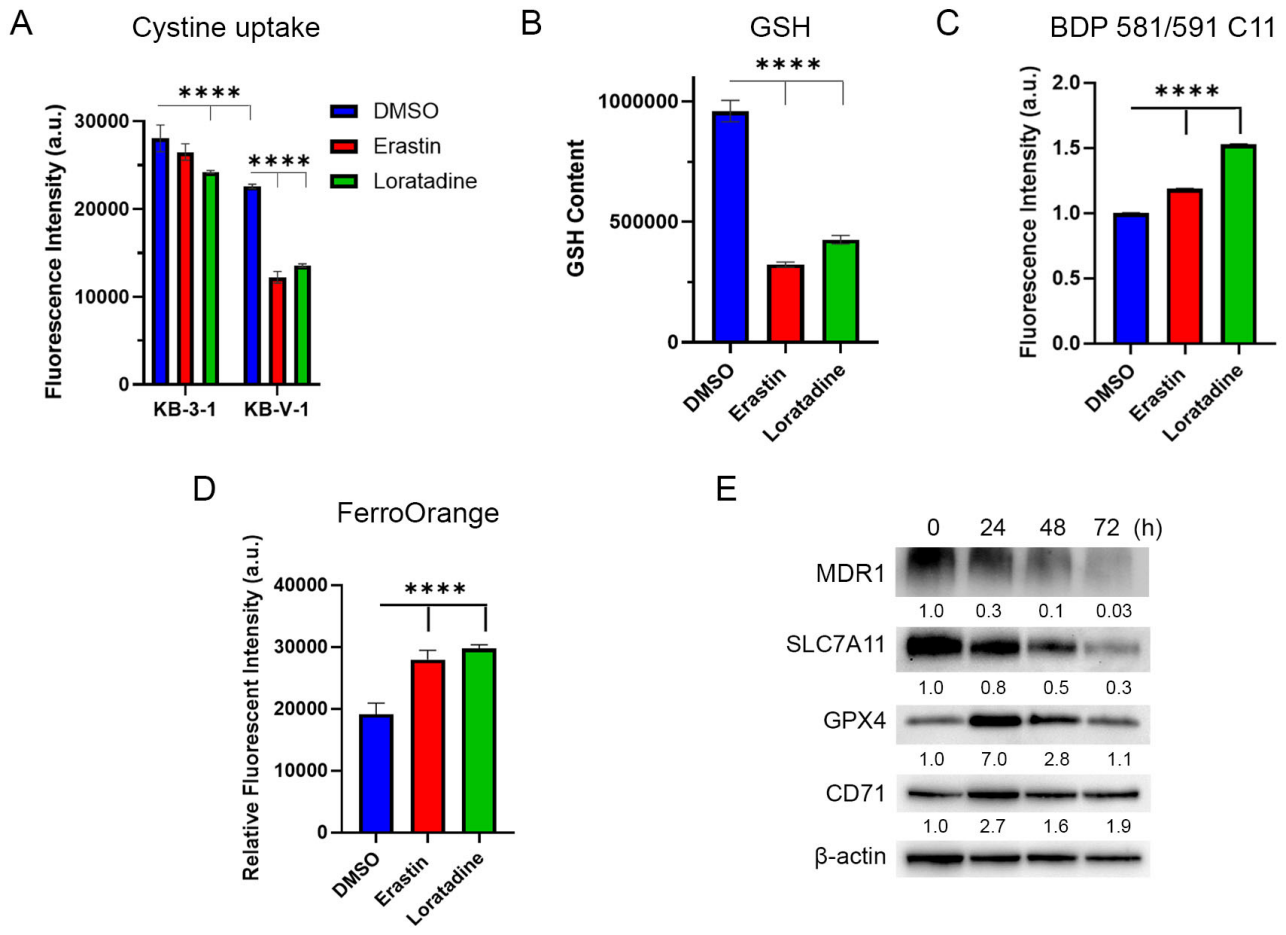
Loratadine inhibits cystine uptake and glutathione synthesis while promoting lipid peroxidation in chemoresistant KB-V-1 cells. **(A)** Cystine uptake in KB-3–1 and KB-V-1 cells following treatment with loratadine (10 µM), erastin (10 µM), or vehicle control. **** *p* < 0.0001. **(B)** Intracellular GSH levels in KB-V-1 cells treated with loratadine (5 µM), erastin (5 µM), or vehicle control for 24 h **** *p* < 0.0001. **(C)** Lipid peroxidation in KB-V-1 cells detected using BODIPY 581/591 C11 following treatment with loratadine (5 µM), erastin (5 µM), or vehicle control for 24 h **** *p* < 0.0001. **(D)** Intracellular ferrous iron (Fe²^+^) levels measured using FerroOrange in KB-V-1 cells treated with loratadine (5 µM), erastin (5 µM), or vehicle control for 24 h **** *p* < 0.0001. **(E)** KB-V-1 cells were treated with loratadine (2 µM), and protein lysates were collected at 0, 24, 48, and 72 (h) Western blot analysis shows the expression levels of MDR1, SLC7A11, GPX4, and CD71.

We further examined the effects of loratadine on ferroptosis-associated pathways in KB-V-1 cells. We treated KB-3–1 and KB-V-1 cells with DMSO, erastin, and loratadine. Loratadine treatment reduced cystine uptake to a level comparable to that observed with erastin in KB-V-1 cells but no significant change in the KB-3–1 chemosensitive cell line (**Figure 2A**). Both loratadine and erastin significantly decreased intracellular GSH levels, indicating suppression of GSH biosynthesis (**Figure 2B**). Loratadine also induced pronounced lipid peroxidation in KB-V-1 cells, comparable to that triggered by erastin (**Figure 2C**). In addition, intracellular Fe²^+^ levels were elevated following treatment with either erastin or loratadine (**Figure 2D**), consistent with the iron-dependent oxidative process characteristic of ferroptosis.

Western blot analysis revealed that loratadine treatment increased the expression of GPX4 and the transferrin receptor 1 (TfR1/CD71), while SLC7A11 expression was transiently upregulated at 24 h but markedly decreased by 72 h (**Figure 2E**). These dynamic molecular changes are consistent with an adaptive cellular response to ferroptotic stress. Additionally, loratadine treatment reduced MDR1/P-glycoprotein expression, suggesting that loratadine may attenuate the multidrug resistant phenotype by suppressing MDR1 levels.

### Loratadine induces ER stress in KB-V-1 cells

**Figure 3:**
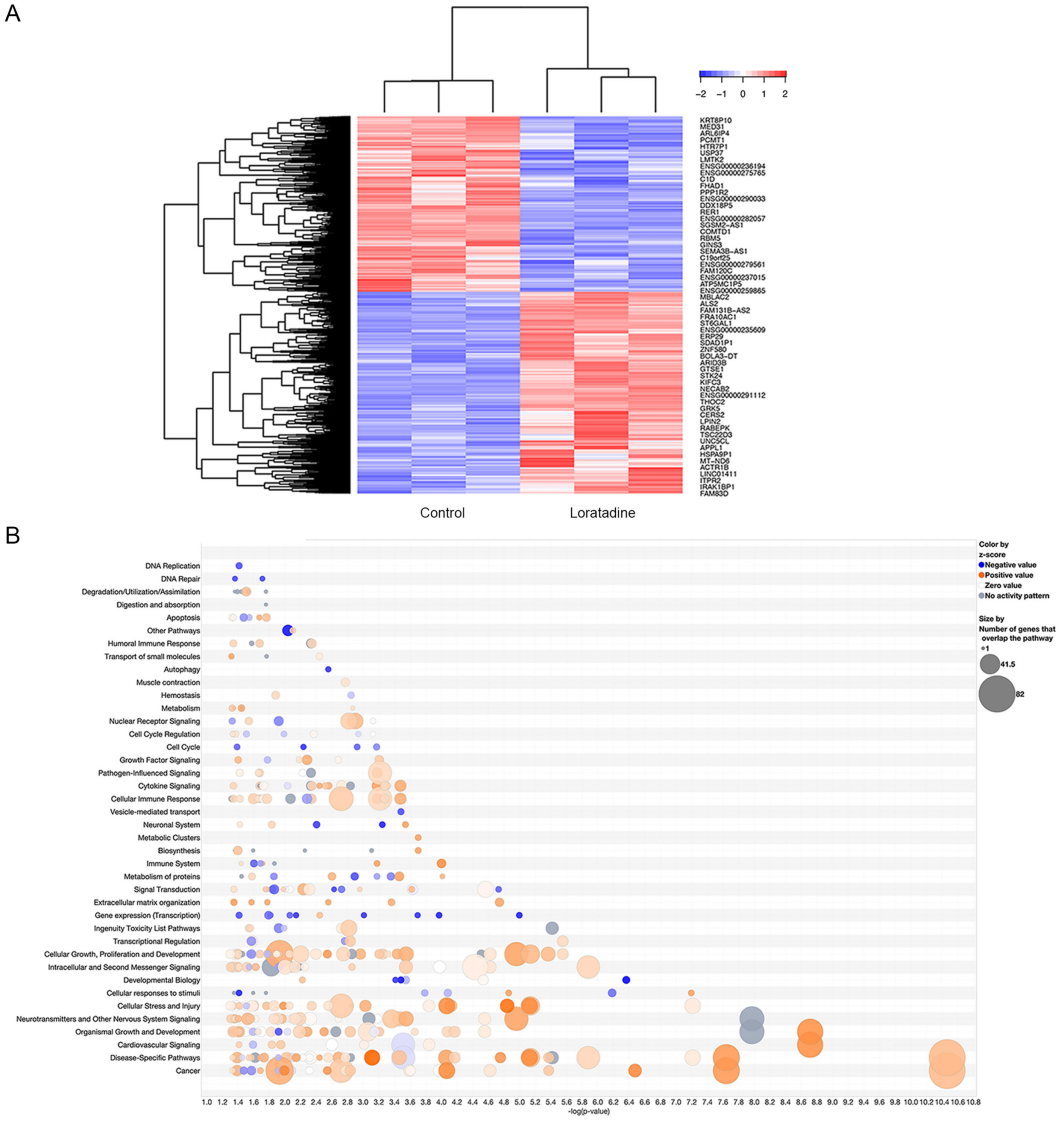
Loratadine induces transcriptomic reprogramming in chemoresistant KB-V-1 cells. **(A)** Heatmap of differentially expressed genes in KB-V-1 cells treated with vehicle or loratadine (2 µM, 24 h). Genes with |log_2_ fold change (Log_2_FC)| > 1.0 and false discovery rate (FDR) *q* < 0.05 were considered significant. Upregulated and downregulated genes are shown in red and blue, respectively. **(B)** Bubble plot of canonical pathways altered by loratadine treatment based on Ingenuity Pathway Analysis (IPA). The y-axis represents canonical pathway categories, and the x-axis indicates the negative log of the Fisher’s exact right-tailed *p*-value. Orange and blue bubbles indicate positive and negative pathway activation scores, respectively.

**Figure 4:**
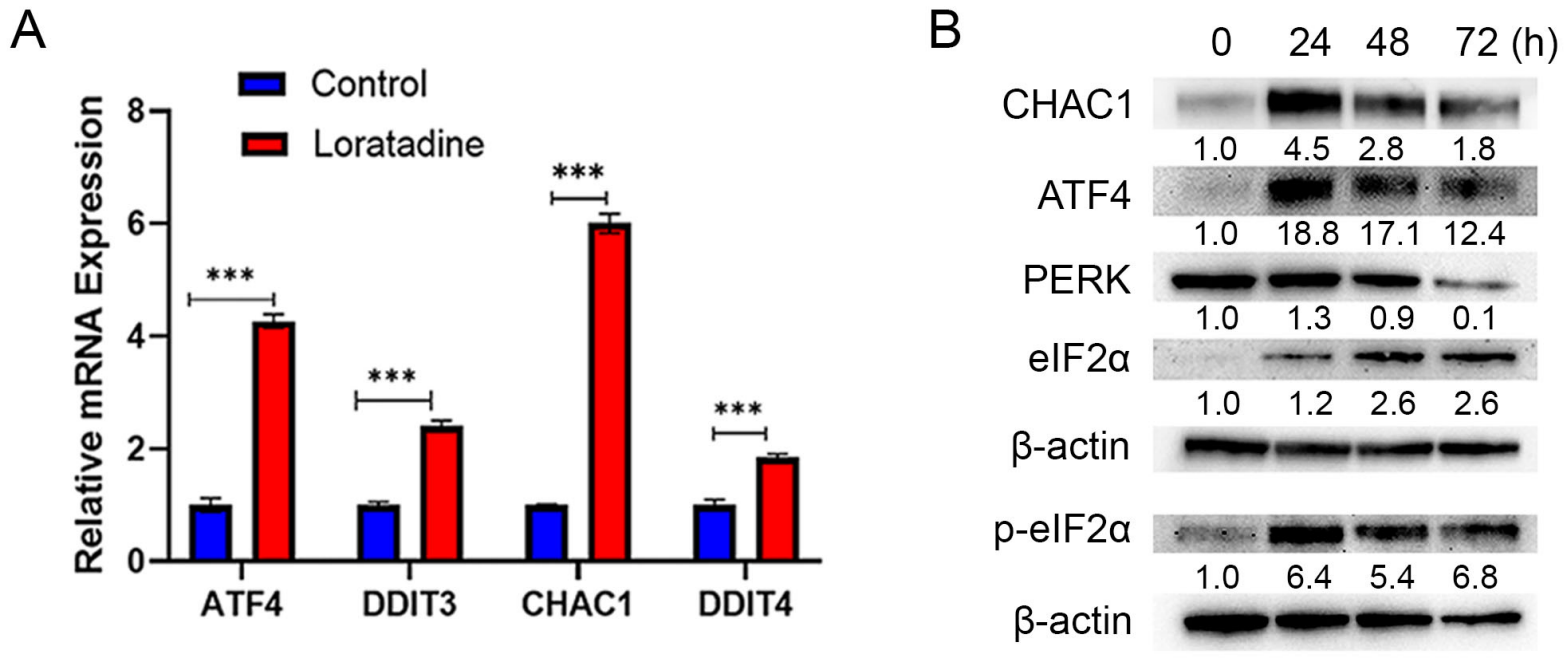
Loratadine modulates the expression of ER stress-related factors in chemoresistant KB-V-1 cells. **(A)** qRT-PCR validation of selected ER-related genes in KB-V-1 cells treated with loratadine (2 µM, 24 h). *** *p* < 0.001. **(B)** Western blot analysis of ER stress-associated proteins in KB-V-1 cells treated with loratadine (2 µM) for 0–72 h.

To gain an unbiased understanding of the molecular targets of loratadine in multidrug resistant cancer cells, we performed RNA sequencing (RNA-seq) analysis of KB-V-1 cells following loratadine treatment (2.0 µM, 24 h). Using a cutoff of absolute Log_2_ fold change (|Log_2_FC|) > 1.0 and a false discovery rate (FDR) q-value < 0.05, loratadine significantly altered the expression of 1,861 unique genes, including 840 upregulated and 1,021 downregulated transcripts (**Figure 3A**). Ingenuity Pathway Analysis (IPA) revealed that these genes were enriched in several stress-related pathways, including activation of activating transcription factor 4 (ATF4) in response to ER stress, PERK-mediated regulation of gene expression, and the response of heme-regulated inhibitor (HRI) to heme deficiency (**Figure 3B**). Among the most prominently upregulated genes were ATF4, CHAC1, DDIT4, ASNS, and GDF15, all of which are associated with the ER stress response and ferroptosis (**Supplementary Figure 1**). These transcriptional changes suggest that loratadine induces ferroptotic cell death at least in part through activation of ER stress pathways. Real-time PCR analysis confirmed that CHAC1, ATF4, DDIT4, and DDIT3 (CHOP) mRNA levels were significantly elevated after 24 h of loratadine treatment (**Figure 4A**).

Western blot analysis further confirmed these findings at the protein level. CHAC1 and ATF4 expression peaked at 24 h and returned to near-baseline levels by 72 h. PERK expression decreased at 72 h, whereas total eIF2α levels increased after 24 h of loratadine exposure. Notably, phosphorylation of eIF2α at serine 51, a canonical marker of PERK pathway activation (33), was significantly upregulated, with maximal induction observed at 24 h (**Figure 4B**). Collectively, these results suggest that loratadine activates the PERK-eIF2α-ATF4 signaling axis, thereby inducing an ER stress response that contributes to ferroptotic cell death in KB-V-1 cells.

### Loratadine inhibits tumor growth *in vivo*

**Figure 5:**
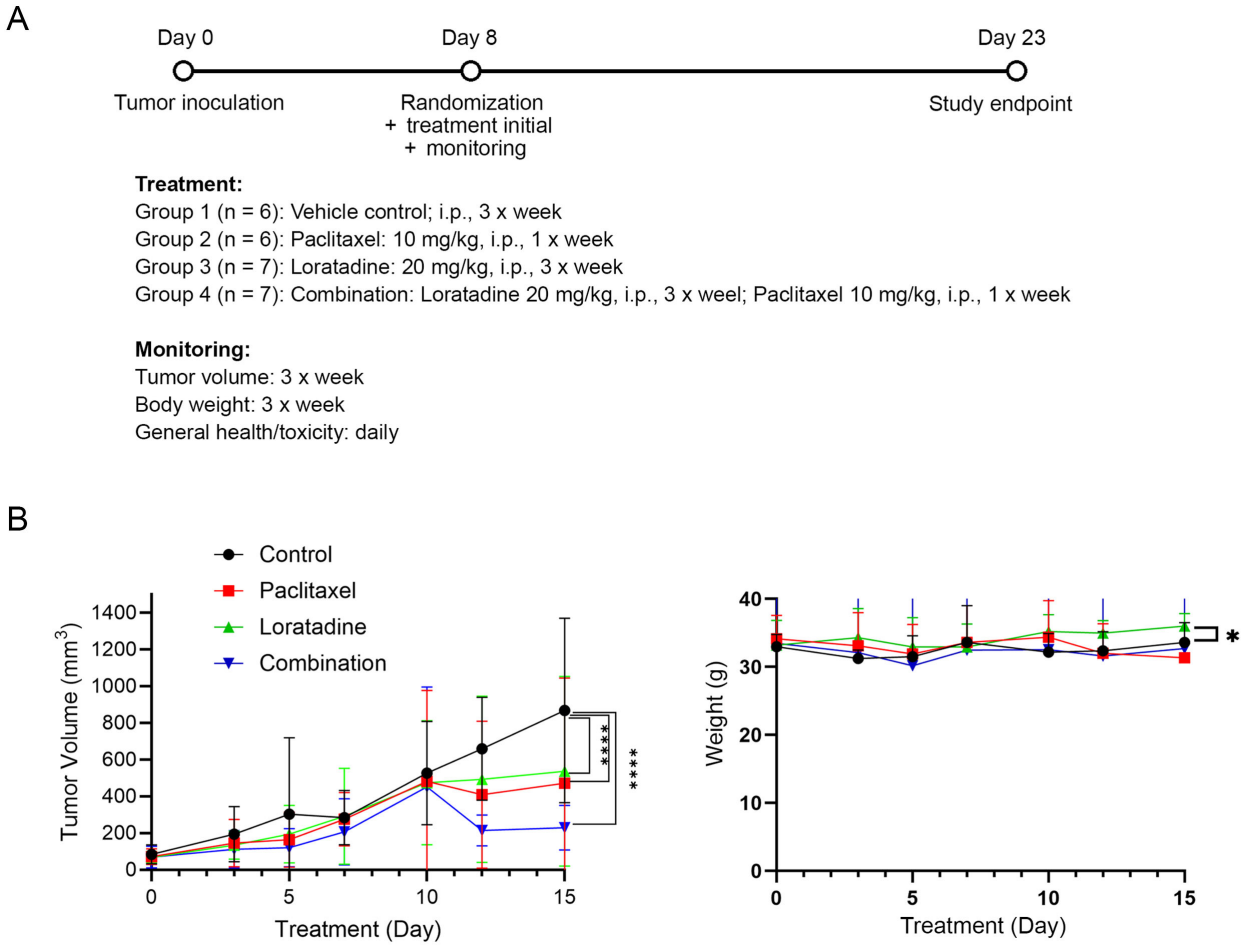
*In vivo* antitumor efficacy of loratadine in the KB-V-1 xenograft model. **(A)** Experimental design and treatment schedule for the animal study. **(B)** Left: tumor growth curves of KB-V-1 xenografts in mice treated with vehicle (n = 6), paclitaxel (10 mg/kg, once weekly; n = 6), loratadine (20 mg/kg, three times per week; n = 7), or the combination of paclitaxel and loratadine (n = 7). All treatments were administered via intraperitoneal injection. Statistical differences in tumor growth among treatment groups were analyzed using two-way ANOVA. **** *p* < 0.0001. Right: average body weights of mice in each treatment group over the course of treatment. * *p* < 0.05.

We next evaluated the antitumor efficacy of loratadine, paclitaxel, and their combination in a multidrug resistant KB-V-1 xenograft model. KB-V-1 cells were inoculated subcutaneously into male athymic nude mice, and treatment was initiated once tumors became established (**Figure 5A**). Compared with vehicle-treated controls (mean tumor volume: 867.80 mm³), intraperitoneal administration of loratadine (20 mg/kg, three times per week) significantly suppressed tumor growth, yielding a tumor growth inhibition (TGI) of 38.15% (mean tumor volume: 536.71 mm³). Paclitaxel monotherapy (10 mg/kg, once per week) achieved a TGI of 45.62% (mean tumor volume: 471.91 mm³). Combination therapy produced the most pronounced antitumor effect, with a TGI of 73.50% (mean tumor volume: 229.98 mm³; **Figure 5B**, left panel), significantly greater than either agent alone (p < 0.05, two-way ANOVA). To assess whether this combination effect exceeded simple additivity, we applied the Bliss independence model (32). The expected additive TGI was 66.37%, yielding a Bliss excess score of +7.13%, indicating a combination effect that is between supra-additive and mildly synergistic. As a supplementary measure, area under the tumor growth curve (AUC) was calculated using the trapezoidal method in GraphPad Prism; while AUC values trended in the expected direction, the differences did not reach statistical significance (p ≥ 0.05), likely reflecting dilution of the integrated area by the pre-divergence period when all groups had comparable tumor volumes early in the study (**Supplementary Figure 2**). Importantly, treatment with loratadine alone or in combination with paclitaxel did not cause significant body weight loss or visible toxicity (**Figure 5B**, right panel). Collectively, these findings indicate that loratadine effectively inhibits the growth of chemoresistant KB-V-1 tumors and enhances the therapeutic efficacy of paclitaxel without increasing systemic toxicity.

## Discussion

**Figure 6:**
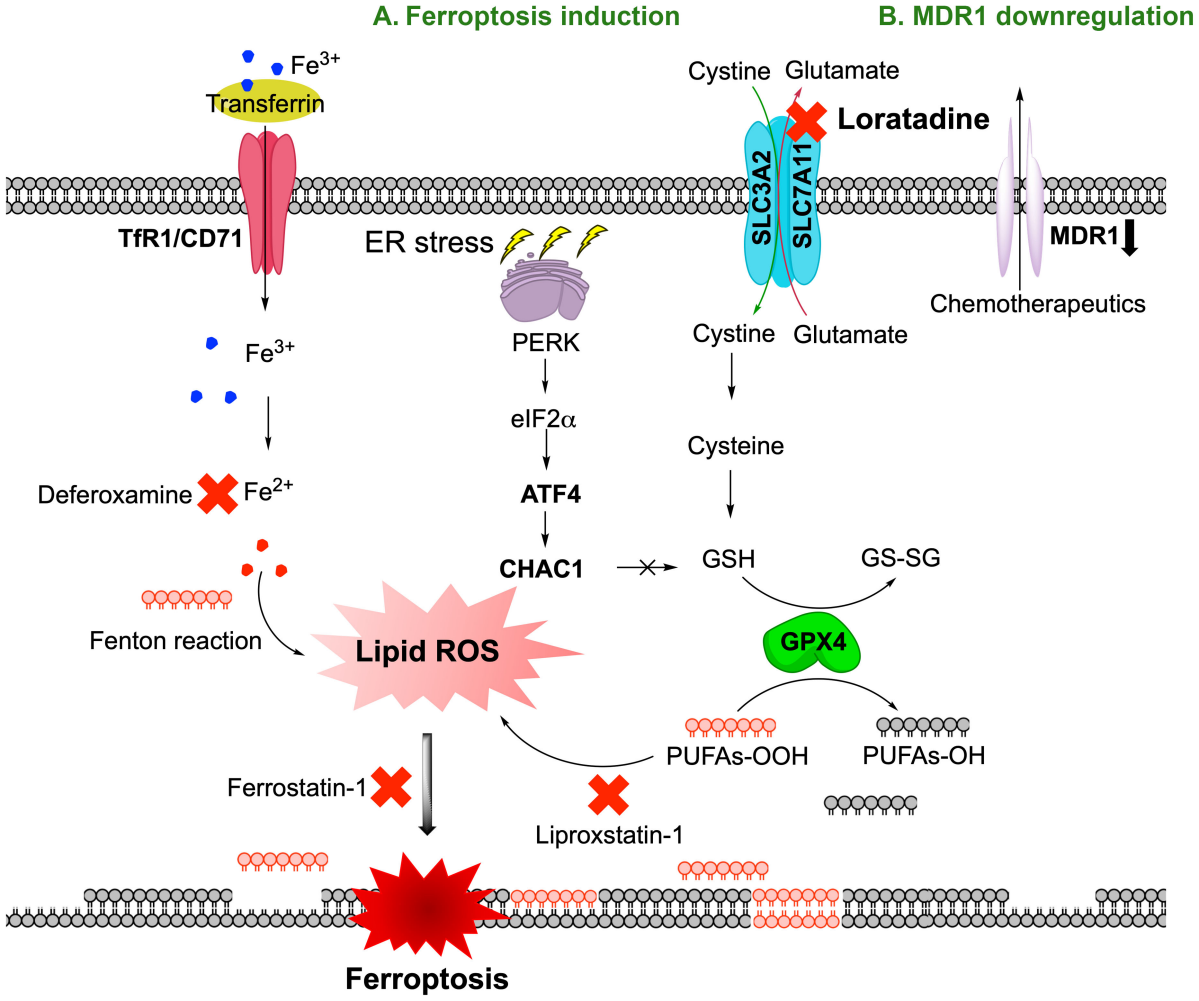
Proposed mechanism of action of loratadine in chemoresistant KB-V-1 cells. Schematic diagram illustrating that loratadine exerts antitumor activity through two complementary mechanisms. **(A)** Ferroptosis induction: loratadine may interact with SLC7A11, leading to reduced cystine uptake, depletion of intracellular GSH, and accumulation of lipid peroxides, thereby inducing ferroptosis. In parallel, loratadine activates ER stress signaling, resulting in upregulation of ATF4 and CHAC1, which further contributes to GSH depletion and ferroptotic cell death. The ferroptosis inhibitors deferoxamine, ferrostatin-1, and liproxstatin-1 attenuate loratadine-induced cell death; **(B)** Suppression of MDR1/P-glycoprotein expression: loratadine downregulates MDR1/P-glycoprotein levels, thereby reducing drug efflux capacity and enhancing the cytotoxicity of chemotherapeutic agents such as paclitaxel. In addition, loratadine may partially induce apoptosis, contributing to its cytotoxic effect. ER, endoplasmic reticulum; GSH: glutathione; GS-SG, glutathione disulfide; PUFAs-OH, hydroxylated polyunsaturated fatty acids; PUFAs-OOH, polyunsaturated fatty acid hydroperoxides; ROS, reactive oxygen species.

Despite advances in cancer treatment, multidrug resistance continues to compromise therapeutic outcomes, leading to clinical failure and relapse. Repurposing FDA-approved drugs with previously unrecognized anticancer properties represents a pragmatic strategy to overcome this challenge. Here, we identify loratadine, a widely prescribed antihistamine, as an agent that targets multidrug resistant cancer cells through ferroptosis induction. Using the KB-V-1 model, we demonstrate that loratadine induces ferroptotic cell death, characterized by increased labile iron, enhanced lipid peroxidation, and GSH depletion. Notably, loratadine also reduces MDR1/P-glycoprotein expression, suggesting that it may modulate features associated with the chemoresistant phenotype in addition to inducing ferroptosis (**Figure 6**). Consistent with our *in vitro* findings, loratadine significantly inhibited tumor growth and prolonged survival in KB-V-1 xenografts without evident systemic toxicity.

Previous studies have reported anticancer effects of loratadine through diverse mechanisms. In colon cancer, loratadine induced DNA damage and activated checkpoint kinase 1 (Chk1), leading to G2/M arrest and enhanced radiosensitivity (34). In non-small-cell lung cancer, loratadine reversed multidrug resistance through apoptosis and lysosomal cell death, while additional studies have implicated inhibition of STAT3 signaling and autophagy-mediated apoptosis (14). Loratadine has also been reported to induce pyroptosis in lung cancer models (12). Epidemiological analyses further suggest an association between antihistamine use and reduced cancer incidence, although the underlying mechanisms remain unclear. Importantly, most prior studies were conducted in drug-sensitive models, and the efficacy of loratadine in clinically relevant chemoresistant settings has remained largely unexplored.

Our findings indicate that KB-V-1 cells are intrinsically vulnerable to ferroptosis. Compared with parental KB-3–1 cells, KB-V-1 cells display reduced basal SLC7A11 expression and heightened sensitivity to erastin, consistent with a constrained redox buffering capacity. Loratadine phenocopies canonical ferroptosis inducers by suppressing cystine uptake, depleting intracellular GSH, increasing labile iron, and promoting lipid peroxidation. The rescue of loratadine-induced cytotoxicity by ferrostatin-1, liproxstatin-1, and deferoxamine, together with the detection of loratadine-induced PARP cleavage and the partial, rather than complete, cytoprotection afforded by the pan-caspase inhibitor Z-VAD-FMK, indicates that ferroptosis is the predominant mode of cell death, with a secondary apoptotic component contributing modestly to the overall cytotoxic response. This pattern is consistent with prior observations in KBV20C cells, where loratadine was also reported to engage apoptotic pathways (19) and supports the emerging view that loratadine can activate multiple cell death modalities, with the dominant mechanism depending on cellular context.

Transcriptomic and protein analyses further revealed activation of the PERK-eIF2α-ATF4 axis, a key component of the integrated stress response linked to ferroptosis regulation (35, 36). Induction of ATF4 and downstream targets, including CHAC1 and DDIT4, supports a model in which loratadine amplifies ER stress-dependent redox collapse. CHAC1-mediated glutathione degradation may further exacerbate GSH depletion, reinforcing lipid peroxidation and ferroptotic commitment (37). The observed GPX4 upregulation likely reflects a compensatory adaptive response to oxidative stress rather than functional ferroptosis resistance, as cell death remained reversible by canonical ferroptosis inhibitors. Together, these findings highlight convergence between ER stress signaling and ferroptotic execution in loratadine-treated cells (**Figure 6**).

A particularly notable finding is the downregulation of MDR1/P-glycoprotein following loratadine exposure. MDR1 overexpression is a well-established mechanism of chemoresistance across multiple cancer types, including KB-V-1 cells (22). In a related multidrug-resistant KB model, KBV20C, loratadine sensitized cells to vincristine despite exhibiting minimal direct P-glycoprotein inhibitory activity, suggesting an alternative mechanism of resistance modulation (19). Importantly, only 2 of 21 histamine receptor antagonists tested conferred this sensitizing activity, indicating that the effect is not a general consequence of histamine receptor inhibition but rather a compound-specific action. These findings suggest that loratadine acts in MDR1-overexpressing cells through a mechanism distinct from its canonical histamine receptor antagonism, consistent with our data showing that ferroptosis induction and MDR1 expression suppression, rather than direct P-glycoprotein efflux inhibition or histamine receptor blockade, are major contributors to its anticancer activity in this chemoresistant setting.

It is important to distinguish between two fundamentally different mechanisms by which P-glycoprotein-mediated resistance may be modulated. Direct P-glycoprotein inhibition, as assessed by Kim et al. (19) and earlier studies (38), refers to competitive or allosteric blockade of transporter efflux function, the mechanism exploited by classical P-glycoprotein inhibitors such as verapamil and tariquidar (39, 40). In contrast, our Western blot data demonstrate that loratadine reduces MDR1/P-glycoprotein protein levels, reflecting a transcriptional, post-transcriptional, or protein-stability regulatory effect that decreases the total cellular capacity for drug efflux rather than directly inhibiting the activity of existing transporter molecules at the cell surface. Thus, functional inhibition of P-glycoprotein efflux activity and downregulation of its protein expression are distinct phenomena and are not in conflict. The reported lack of direct P-glycoprotein inhibitory activity by loratadine (19) is therefore fully consistent with, and complementary to, our proposed mechanism of resistance modulation through MDR1 expression suppression.

Consistent with this concept, our data show reduced MDR1 expression following loratadine treatment, which may enhance intracellular retention of chemotherapeutic substrates and contribute to chemosensitization. Although the precise mechanism underlying MDR1 suppression remains to be defined, reduced P-glycoprotein levels would be expected to increase intracellular retention of paclitaxel, potentially contributing to the enhanced antitumor efficacy observed with combination treatment. Supporting this broader concept, recent work has shown that simultaneous ferroptosis activation and modulation of drug-resistance pathways can enhance therapeutic responses in resistant cancer models (41).

Several mechanistic questions warrant further investigation. The direct molecular interaction between loratadine and the SLC7A11-GSH-GPX4 axis remains to be established, and the regulatory basis for MDR1 downregulation requires clarification. Future studies employing genetic manipulation of SLC7A11 or MDR1, as well as functional efflux assays, will be necessary to define causality. Additionally, determining whether loratadine selectively targets specific tumor subtypes with heightened redox dependency or cooperates with other redox-active therapies will further refine its translational potential.

Although ferroptosis inducers such as erastin, sulfasalazine, and sorafenib have demonstrated preclinical promise, their clinical application has been limited by pharmacokinetic constraints and dose-limiting toxicities (4, 42–49). Moreover, MDR1/P-glycoprotein-mediated drug efflux has recently been shown to compromise the efficacy of certain ferroptosis-inducing agents (50), further narrowing their therapeutic utility in multidrug resistant cancers. Identifying clinically tolerable agents capable of inducing ferroptosis in therapy-resistant settings therefore remains an important unmet need. Given its established safety profile, favorable oral bioavailability, and decades of widespread clinical use, loratadine is particularly attractive as a repurposed candidate for oncology. Collectively, our findings demonstrate that loratadine perturbs redox homeostasis and activates ferroptotic signaling in resistant cancer cells while simultaneously suppressing MDR1/P-glycoprotein expression. Repurposing loratadine may therefore provide a practical strategy for integrating ferroptosis-based approaches into resistance-directed cancer therapy.

## Data Availability

The datasets presented in this study can be found in online repositories. The names of the repository/repositories and accession number(s) can be found below: https://www.ncbi.nlm.nih.gov/, PRJNA1423803.
