# Supplementary material for: Repurposing loratadine to induce ferroptosis and overcome multidrug resistance: preclinical evidence in KB-V-1 cells
Source: Front Oncol. 2026 Jun 17;16:1829405. doi: 10.3389/fonc.2026.1829405 (PMC13318782; doi:10.3389/fonc.2026.1829405)
Supplement: Supplementary file 3 [file Table3.docx]

Ovid MEDLINE(R) ALL <1946 to June 18, 2025>

1 Radiotherapy/ 44884

2 Radiation Oncology/ 6322

3 exp Neoplasms/rt [Radiotherapy] 203601

4 Proton Therapy/ 6093

5 (Radiotherapy or "radiation therapy" or "External beam" or EBRT or "proton therapy" or "proton beam therapy" or IMRT or IGRT or "stereotactic radiosurgery" or SABR or SBRT or telecobalt or linac*).ab,ti,hw,kf. 383901

6 or/1-5 440340

7 Health Services Accessibility/ 93702

8 Health Equity/ 5269

9 Health Inequities/ 835

10 Healthcare Disparities/ 26683

11 Health status disparities/ 22082

12 Right to health/ 341

13 Social Determinants of Health/ 8972

14 socioeconomic disparities in health/ 410

15 ((access* or inaccess* or equit* or equal* or unequal* or inequal* or disparit* or barrier* or obstacle* or challeng* or utili#ation or avail* or convenien* or delay* or "wait-time*" or capacit* or "social determinant*" or "right to health*" or gap* or expan*) adj4 (health or "health care" or healthcare or treatment* or therap* or radiotherap* or service*)).ab,ti,hw,kf. 724174

16 ((global or equit* or equal* or expan* or democratiz* or democratis*) adj3 access*).ab,ti,hw,kf. 22437

17 or/7-16 734306

18 Developing Countries/ 84139

19 Medically Underserved Area/ 7747

20 Vulnerable Populations/ 13790

21 "Health Disparate Minority and Vulnerable Populations"/ 39

22 (disadvantage* or vulnerab* or racial* or impoverished or marginali* or underserved or underrepresented).ab,ti,hw,kf. 477151

23 poverty areas/ 6848

24 Rural Population/ 74712

25 (rural* or remote* or northern).ab,ti,hw,kf. 576651

26 (underserved or "under served" or "low resource*" or "limited resource" or "resource-limited" or "resource-constrained" or "resource scarce" or "under-resourced").ab,ti,hw,kf. 62674

27 Africa/ or "Africa South of the Sahara"/ or exp Africa, Central/ or exp Africa, Eastern/ or exp Africa, Northern/ or exp Africa, Southern/ or exp Africa, Western/ 355451

28 americas/ or exp caribbean region/ or exp central america/ or latin america/ or exp south america/ 279500

29 exp Asia, Northern/ or exp Asia, Central/ or exp Asia, Southern/ or Asia/ or exp Asia, Southeastern/ or exp Asia, Eastern/ or exp Asia, Western/ 1148522

30 (afghan* or africa* or albania* or algeria* or angola* or antigua* or barbuda* or argentin* or armenia* or aruba* or azerbaijan* or bahrain* or bangladesh* or bengal* or bangal* or barbados* or barbadian* or bajan or bajans or belarus* or belorus* or byelarus* or byelorus* or belize* or benin* or dahomey or bhutan* or bolivia* or bosnia* or herzegovin* or botswan* or batswan* or bechuanaland* or brazil* or brasil* or bulgaria* or burkina* or burkinese* or "upper volta*" or burundi* or urundi* or "cabo verde*" or "cape verde*" or cambodia* or kampuchea* or khmer* or cameroon* or cameroun* or "ubangi shari*" or chad* or chile* or china* or chinese or colombia* or comoro* or comore* or comorian* or mayotte* or congo* or zaire* or "costa rica*" or "cote d'ivoir*" or "cote d' ivoir*" or "cote divoir*" or "cote d ivoir*" or "ivory coast*" or ivorian* or croatia* or cuba or cuban or cubans or "cuba's" or cyprus* or cypriot* or czech* or djibouti* or "french somaliland*" or dominica* or ecuador* or egypt* or "united arab republic*" or "el salvador*" or salvadoran* or guinea* or equatoguinea* or eritrea* or estonia* or eswatini* or swaziland* or swazi* or swati* or ethiopia* or fiji* or gabon* or gabonese* or gabonaise* or gambia* or ((georgia or georgian or georgians) not (atlanta or california or florida)) or ghana* or gibraltar* or greece* or greek* or grecian* or grenada* or grenadian* or guam* or guatemala* or guyana* or guiana* or guyanese* or haiti* or hispaniola* or hondura* or hungary* or hungarian* or india* or indonesia* or iran* or iraq* or "isle of man*" or jamaica* or jordan* or kazakh* or kenya* or karabati* or korea* or kosovo* or kosova* or kyrgyz* or kirgiz* or kirghiz* or laos or lao or laotian* or latvia* or lebanon* or lebanese* or lesotho* or lesothan* or lesothonian* or basutoland* or mosotho* or basotho* or liberia* or libya* or jamahiriya* or lithuania* or macedonia* or madagasca* or malagasy* or malawi* or nyasaland* or malaysia* or malay* federation or maldives* or maldivian* or "indian ocean" or mali or malian* or "mali's" or malta or maltese* or "malta's" or micronesia* or marshallese* or kiribati* or "marshall island*" or nauru or nauran or nauruans or "naurian's" or mariana or marianas or palau or paluan* or tuvalu* or mauritania* or mauritan* or mauritius* or mexico* or mexican* or moldova* or moldovia* or mongol* or montenegr* or morocco* or moroccan* or ifni or mozambique* or mozambican* or myanmar* or burma* or burmese or namibia* or nepal* or "new caledonia*" or "netherlands antill*" or nicaragua* or niger* or oman or omani or omanis or "oman's" or pakistan* or palestin* or gaza* or "west bank*" or panama* or paraguay* or peru or peruvian* or "peru's" or philippine* or philipine* or phillipine* or phillippine* or filipino* or filipina* or poland* or polish or pole or poles or portugal* or portuguese or "puerto ric*" or romania* or russia* or ussr* or soviet* or rwanda* or rwandese or ruanda* or ruandese or samoa* or "navigator island*" or "pacific island*" or polynesia* or "sao tome and principe*" or "sao tomean*" or santomean* or "saudi arabia*" or saudi or saudis or senegal* or serbia* or seychell* or "sierra leone*" or slovak* or sloven* or melanesia* or "solomon island*" or "norfolk island*" or somali* or "sri lanka*" or ceylon* or "saint kitts and nevis*" or "st kitts and nevis*" or kittian* or nevisian* or "saint lucia*" or "st lucia*" or "saint vincent*" or "st vincent*" or vincentian* or grenadine* or sudan* or surinam* or syria* or tajik* or tadjik* or tadzhik* or tanzania* or tanganyika* or thai* or "timor leste*" or "east timor*" or timorese* or togo or togoles* or "togo's" or tonga* or trinidad* or tobago* or tunisia* or turkiy* or turkey* or turk or turks or turkish or turkmen* or uganda* or ukrain* or uruguay* or uzbek* or vanuatu* or "new hebrides*" or venezuela* or vietnam* or "viet nam*" or yemen* or yugoslav* or zambia* or zimbabwe* or rhodesia* or "arab* countr*" or "middle east*" or "global south" or sahara* or subsahara* or magreb* or maghrib* or "west indies*" or caribbean* or "central america*" or "latin america*" or "south america*" or "central asia*" or "north asia*" or "northern asia*" or "southeastern asia*" or "south eastern asia*" or "southeast asia*" or "south east asia*" or "west asia*" or "western asia*" or "east europe*" or "eastern europe*").ab,ti,hw,kf. 3528564

31 ("developing countr*" or "developing nation*" or "developing population*" or "developing world" or "less developed countr*" or "less developed nation*" or "less developed world" or "lesser developed countr*" or "lesser developed nation*" or "lesser developed world" or "under developed countr*" or "under developed nation*" or "under developed world" or "underdeveloped countr*" or "underdeveloped nation*" or "underdeveloped world" or "middle income countr*" or "middle income nation*" or "middle income population*" or "low income countr*" or "low income nation*" or "low income population*" or "lower income countr*" or "lower income nation*" or "lower income population*" or "underserved countr*" or "underserved nation*" or "underserved population*" or "under served population*" or "under served nation*" or "under served population*" or "geographic* underserved" or "geographic* under served" or "deprived countr*" or "deprived population*" or "high burden countr*" or "high burden nation*" or "countdown countr*" or "countdown nation*" or "poor countr*" or "poor nation*" or "poor population*" or "poor world" or "poorer countr*" or "poorer nation*" or "poorer population*" or "poorer world" or "developing econom*" or "less developed econom*" or "underdeveloped econom*" or "under developed econom*" or "middle income econom*" or "low income econom*" or "lower income econom*" or "low gdp" or "low gnp" or "low gross domestic" or "low gross national" or "lower gdp" or "lower gnp" or "lower gross domestic" or "lower gross national" or "low- and middle-income" or lmic or lmics or "third world" or "lami countr*" or "transitional countr*" or "emerging econom*" or "emerging nation*").ti,ab,hw,kf. 227336

32 or/18-31 4583138

33 6 and 17 and 32 2554
